# Ageing is associated with maladaptive immune response and worse outcome after traumatic brain injury

**DOI:** 10.1093/braincomms/fcac036

**Published:** 2022-02-16

**Authors:** Federico Moro, Francesca Pischiutta, Anaïs Portet, Edward J. Needham, Emma J. Norton, John R. Ferdinand, Gloria Vegliante, Eliana Sammali, Rosaria Pascente, Enrico Caruso, Edoardo Micotti, Daniele Tolomeo, Rafael di Marco Barros, Erik Fraunberger, Kevin K. W. Wang, Michael J. Esser, David K. Menon, Menna R. Clatworthy, Elisa R. Zanier

**Affiliations:** 1 Laboratory of Acute Brain Injury and Therapeutic Strategies, Department of Neuroscience, Istituto di Ricerche Farmacologiche Mario Negri IRCCS, 20156 Milan, Italy; 2 Molecular Immunity Unit, Department of Medicine, Laboratory of Molecular Biology, University of Cambridge, Cambridge CB2 0QH, UK; 3 Division of Anaesthesia, University of Cambridge, Addenbrooke’s Hospital, Cambridge CB2 0QH, UK; 4 Neuroscience Intensive Care Unit, Department of Anesthesia and Critical Care, Fondazione IRCCS Cà Granda Ospedale Maggiore Policlinico, 20122 Milan, Italy; 5 Laboratory of Biology of Neurodegenerative Disorders, Department of Neuroscience, Istituto di Ricerche Farmacologiche Mario Negri IRCCS, 20156 Milan, Italy; 6 Cumming School of Medicine, Alberta Children’s Hospital Research Institute, Hotchkiss Brain Institute, University of Calgary, Calgary, AB, Canada; 7 Program for Neurotrauma, Neuroproteomics and Biomarker Research, Departments of Emergency Medicine, Psychiatry and Neuroscience, University of Florida, Gainesville, FL, USA

**Keywords:** traumatic brain injury, ageing, neuroinflammation, meninges, reactive astrogliosis

## Abstract

Traumatic brain injury is increasingly common in older individuals. Older age is one of the strongest predictors for poor prognosis after brain trauma, a phenomenon driven by the presence of extra-cranial comorbidities as well as pre-existent pathologies associated with cognitive impairment and brain volume loss (such as cerebrovascular disease or age-related neurodegeneration). Furthermore, ageing is associated with a dysregulated immune response, which includes attenuated responses to infection and vaccination, and a failure to resolve inflammation leading to chronic inflammatory states. In traumatic brain injury, where the immune response is imperative for the clearance of cellular debris and survey of the injured milieu, an appropriate self-limiting response is vital to promote recovery. Currently, our understanding of age-related factors that contribute to the outcome is limited; but a more complete understanding is essential for the development of tailored therapeutic strategies to mitigate the consequences of traumatic brain injury. Here we show greater functional deficits, white matter abnormalities and worse long-term outcomes in aged compared with young C57BL/6J mice after either moderate or severe traumatic brain injury. These effects are associated with altered systemic, meningeal and brain tissue immune response. Importantly, the impaired acute systemic immune response in the mice was similar to the findings observed in our clinical cohort. Traumatic brain-injured patient cohort over 70 years of age showed lower monocyte and lymphocyte counts compared with those under 45 years. In mice, traumatic brain injury was associated with alterations in peripheral immune subsets, which differed in aged compared with adult mice. There was a significant increase in transcription of immune and inflammatory genes in the meninges post-traumatic brain injury, including monocyte/leucocyte-recruiting chemokines. Immune cells were recruited to the region of the dural injury, with a significantly higher number of CD11b^+^ myeloid cells in aged compared with the adult mice. In brain tissue, when compared with the young adult mice, we observed a more pronounced and widespread reactive astrogliosis 1 month after trauma in aged mice, sustained by an early and persistent induction of proinflammatory astrocytic state. These findings provide important insights regarding age-related exacerbation of neurological damage after brain trauma.

## Teaser

Moro *et al*. report an exacerbated systemic, meningeal and brain tissue immune response to traumatic brain injury in aged subjects, contributing to worse behavioural and anatomical outcomes. These results will help designing tailored therapies to mitigate the consequences of brain trauma in the elderly.

## Graphical Abstract

**Graphical abstract:**
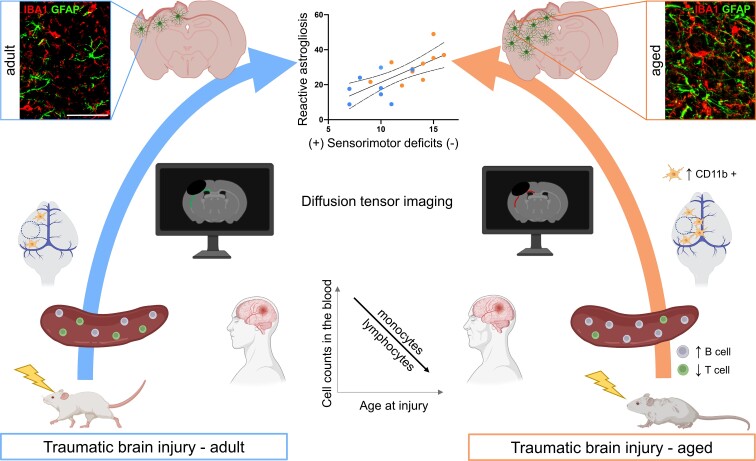

## Introduction

The global population aged 60 years or over grew from 382 million in 1980 to 962 million in 2017 and is estimated to reach 2 billion by 2050,^1^ with profound implications for the planning and delivery of health and social care. Ageing increases the risk of traumatic brain injury (TBI).^2^ A recent large European study^3^ showed a demographic change of TBI, characterized by the older patients (28% aged >65) with more comorbidities and injuries more frequently caused by falls compared with the previous observational studies.^4,5^ Age is a strong predictor of mortality and unfavourable outcome after TBI, with substantial increases in the odds of death and poor neurologic outcome after the age of 70.^6^ These data indicate that TBI should no longer be considered mainly a disease of otherwise healthy young patients and should motivate research on TBI in the underserved aged population.

Several studies have highlighted the significance of age in worsening TBI severity, structural damage and neurological outcome. During ageing, there is a gradual accumulation of pathologies associated with physical decline, cognitive impairment and brain volume loss. The adverse biological effects of normal ageing include cellular senescence, accumulation of DNA damage and misfolded proteins, impaired debris clearance and increased oxidative stress. The old age increases microglial senescence and secondary neuroinflammation, and worsens neurological outcomes after acute TBI in mice.^7^ Furthermore, decreased systemic or local immunological competence,^8,9^ and meningeal lymphatic dysfunction,^10^ can impede TBI recovery and contribute to poor prognosis in the elderly. While the aged brains may react differently to TBI than the young ones, the distinct biological mechanisms that make the aged brain more susceptible to damage are poorly investigated and need to be further characterized. A better understanding of the age-related factors contributing to poor recovery would inform the development of tailored therapeutic strategies to mitigate the consequences of TBI in older patients.

The aim of this work was to investigate the effect of ageing on functional and structural consequences of moderate TBI (mTBI) and severe TBI (sTBI) by analysing behavioural outcomes and pathological damage. Ageing, on its own, results in immune cells with a ‘primed’ phenotype, which is characterized by an exaggerated and uncontrolled inflammatory response to an immune stimulus. We hypothesized that TBI represents a second hit to such primed immune cells, precipitating injury progression in aged subjects. We provide data from TBI in patients and a mouse model to address this hypothesis in systemic, meningeal and brain tissue inflammation.

## Materials and methods

### Study approval

The IRFMN adheres to the principles set out in the following laws, regulations and policies governing the care and use of laboratory animals: Italian Governing Law (D.lgs 26/2014; Authorization n.19/2008-A issued 6 March 2008 by Ministry of Health); Mario Negri Institutional Regulations and Policies providing internal authorization for persons conducting animal experiments (Quality Management System Certificate—UNI EN ISO 9001:2008—Reg. No. 8576-A); the NIH Guide for the Care and Use of Laboratory Animals (2011 edition) and EU directives and guidelines (EEC Council Directive 2010/63/UE). They were reviewed and approved by the Mario Negri Institute Animal Care and Use Committee that includes *ad hoc* members for ethical issues, and by the Italian Ministry of Health (Decreto no. D/07/2013-B and 301/2017-PR).

Human subjects were recruited at Cambridge University Hospital, UK, through studies approved by the Cambridgeshire Research Ethics Committee (REC 97/290 and REC 17/EE/0025).

### Mice

Young adult (9–10 weeks of age, referred to as ‘adult’) and aged (15–18 months of age) C57BL/6J male mice (Envigo RMS srl) were used. They were housed in a pathogen-free vivarium with constant temperature (21 ± 1°C) and relative humidity (60 ± 5%), a 12 h light–dark cycle and free access to pellet food and water. All animal experiments were designed in accordance with the ARRIVE guidelines,^11^ with a commitment to refinement, reduction and replacement, minimizing the numbers of mice and using biostatistics to optimize mouse numbers (as in our previous work with the mouse TBI model).^12–14^ Thus, for the statistical validity, we used 8–10 mice for the behavioural tests, and 4–8 mice for histology and genes analysis. The mice were assigned to the different experimental groups using randomization lists (www.randomizer.org). Behavioural, imaging, histological assessments and gene analysis were done by researchers blinded to the experimental groups.

### Study design

**Figure 1:**
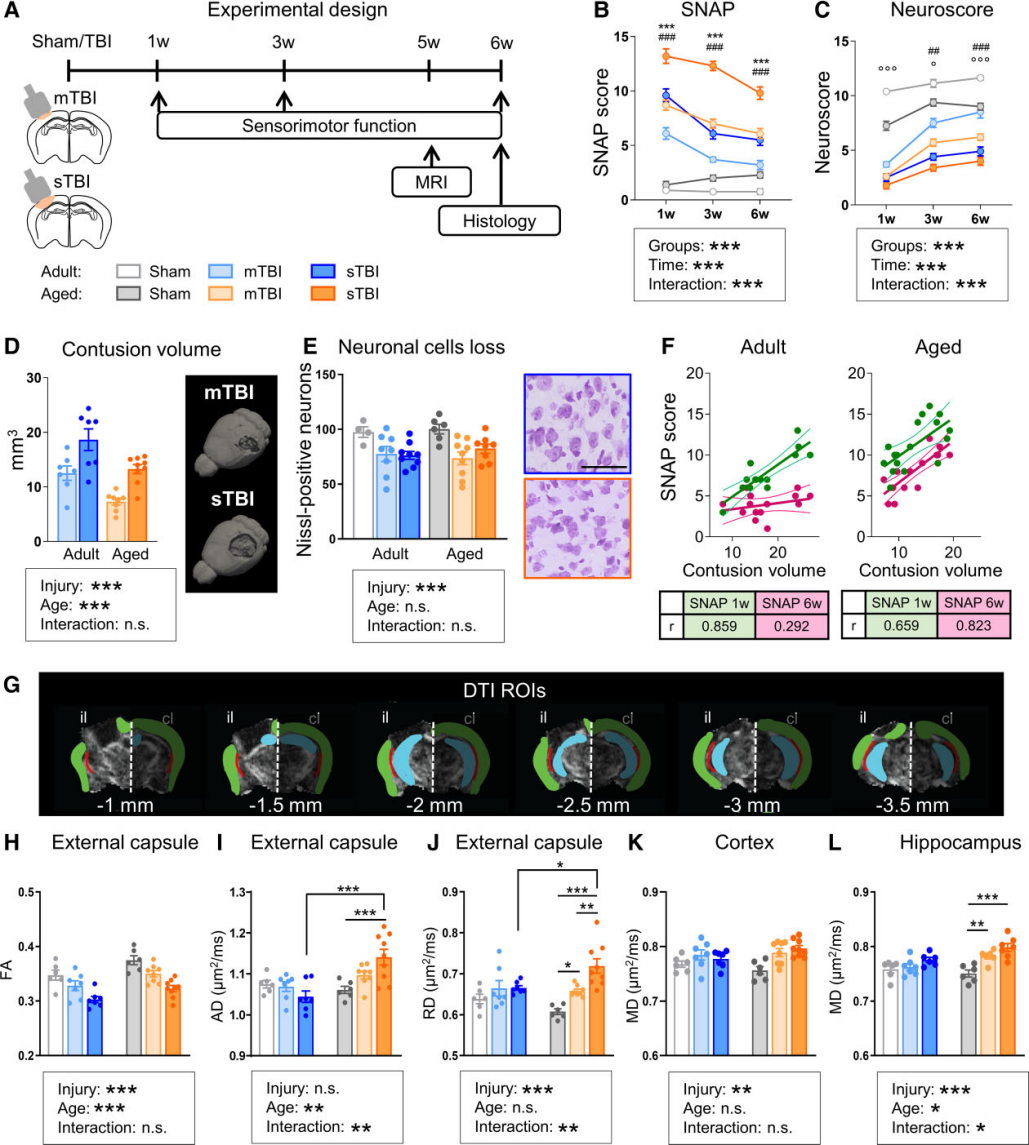
**Age-related differences in behavioural deficits, anatomical damage and DTI parameters after TBI.** (**A**) Experimental design. Assessment of sensorimotor deficits by SNAP (**B**) and neuroscore (**C**) tests (adult and aged: sham *n* = 8, mTBI *n* = 10, sTBI *n* = 10). (**D**) Representative 3D reconstruction and quantification of anatomical damage, by T2w-MRI at 5 weeks after injury. (**E**) Representative Nissl-stained cortical areas of adult and aged TBI mice 6 weeks after injury and relative quantification. (**F**) Pearson correlations between contusion volume and SNAP scores at 1 (green) and 6 weeks (purple) in adult and aged mice. (**G**) The rostrocaudal ROI selection for external capsule (EC, red), cortex (green) and hippocampus (blue) over six coronal slices 0.5 mm of thick are shown. Quantification of fractional anisotropy (FA) (**H**), axial diffusivity (**I**) and radial diffusivity (**J**) in the EC or mean diffusivity in the cortex (**K**) or hippocampus (**L**). Data are mean ± SEM. Each data point refers to a single animal. Bar 50 μm. Two-way RM-ANOVA, Tukey's *post hoc*: °*P* < 0.05, °°°*P* < 0.001 sham adult versus aged; ##*P* < 0.01, ###*P* < 0.001 mTBI adult versus aged, ***P < 0.001 sTBI adult versus aged (**B** and **C**) or two-way ANOVA, Tukey’s *post hoc*: *P < 0.05, **P < 0.01, ***P < 0.001 (**D**, **E**, **H**-**L**). Significant group effects of injury, age and interaction are shown in each box.

To explore the effects of ageing and injury severity on outcomes, adult and aged mice were exposed to either sham, mTBI or sTBI and assessed for behavioural outcome longitudinally up to 6 weeks, and for structural outcome by MRI and histopathology at 5 and 6 weeks, respectively (Fig. 1A). Imaging studies and evaluation of reactive astrogliosis in this first set of experiments showed greater age-related differences in sTBI compared with mTBI mice. Thus, also in compliance with the ‘reduction’ principle of 3Rs for animal research, we restricted further studies aimed at characterizing the inflammatory response to the sTBI cohorts. We next explored the effects of age on TBI-induced inflammation. We analysed (i) systemic inflammation 3 days post-TBI in mice by flow cytometry; (ii) systemic inflammation 1 week post-TBI in patients using automated five-part differentiation haematology data gathered as part of standard clinical care; (iii) immune meningeal response to TBI by bulk RNA sequencing and age-related meningeal immune infiltration by histopathology 3 days and 6 weeks post-TBI in mice; (iv) age-related inflammatory activation in sham and sTBI on the functional commitment of glial at 3 or 7 days by real-time RT–PCR in mice.

### Experimental brain injury

Mice were anaesthetized with isoflurane inhalation (Merial, Italy) (induction: 3–4%, maintenance: 1.5–2%) in an N_2_O/O_2_ (70%/30%) mixture, then placed in a stereotaxic frame. Mice were subjected to craniectomy (in case of a dural breach, mice were excluded) followed by induction of a single lateral controlled cortical impact brain injury over the left parietotemporal cortex, as previously described.^15^ Briefly, the injury was induced by a 3 mm rigid impactor (tip size) driven by a pneumatic piston, rigidly mounted at an angle of 20° from the vertical, and applied perpendicularly to the surface of the exposed dura mater, between the bregma and lambda over the left parietotemporal cortex (AP −2.5 mm, M L −2.5 mm) at a velocity of 5 m/s. The deformation depth was either 0.5 or 1 mm, resulting, respectively, in mTBI or sTBI. After the injury, the craniotomy was covered with a cranioplasty and the scalp sutured. Sham mice received identical anaesthesia, skin incision and suture, but were not subjected to craniotomy and brain injury. During all surgical procedures, mice were maintained at a body temperature of 37°C.

### Behavioural tests

#### Simple neuroassessment of asymmetric impairment

Mice were tested as described,^14,16^ to assess neurological parameters including vision, proprioception, motor strength and posture. The eight tests evaluate: interaction with the handler, grip strength, visual placing, pacing/circling, gait and posture, head tilt, visual field, coordination and proprioception. Scores range from 0 (normal) to 5 (severely impaired) for each test and were summed to give an overall score that ranges from 0 (best) to 40 (worst).

#### Neuroscore

Mice were scored from 4 (normal) to 0 (severely impaired) for each of the following indices: (i) forelimb function while walking on the grid, and flexion function response during suspension by the tail; (ii) hindlimb function while walking on the grid and extension function during suspension by the tail and (iii) resistance to lateral right and left pulsion. The scores obtained in each test were summed to give an overall score that ranges from 0 (worst) to 12 (best).^13,17^

### MRI studies

At 5 weeks post-TBI, mice were anaesthetized with isoflurane inhalation (maintenance 1.5%) in an N_2_O/O_2_ (70%/30%) mixture. Imaging was done on a 7 T small-bore animal scanner (Bruker Biospec, Ettlingen, Germany). Two actively decoupled radio frequency (RF) coils were used: a volume coil of 7.2 cm diameter used as the transmitter and a surface coil as the receiver.^13,18^

#### MRI data acquisition

Anatomical changes, evaluated in Coronal, 2D, T_2_-weighted (T2w) RARE sequences obtained with a voxel size of FOV 1.5 × 1.5 cm, matrix150 × 150, 37 slices with a thickness of 300 μm, repetition time (TR) 5500 ms, echo time (TE) 66 ms, RARE factor 8 and number of averages 12.

Diffusion tensor data were acquired in coronal diffusion tensor imaging (DTI), echo planar imaging (EPI) sequences with an in-plane image resolution of 117 × 156 μm (field of view 1.5 × 1.5 cm^2^, matrix128 × 96, slice thickness 0.5 mm); TR 4500 ms, TE 32 ms. Four averages were used to increase the signal-to-noise ratio. For diffusion encoding *b*, factors of 800 mm^2^/s were applied along 19 isotropic directions of the 3D space.

#### MRI data analysis

The diffusion tensor was computed using the FSL software https://fsl.fmrib.ox.ac.uk/fsl/fslwiki/FSL. The software reconstructs the diffusion tensor generating the three eigenvalues λ1, λ2, λ3 maps and the associated eigenvectors (i.e. the three principal diffusion directions) determining fractional anisotropy (FA), axial diffusivity (AD), radial diffusivity (RD) and mean diffusivity (MD) as described.^13^ Regions of interest (ROIs) were the external capsule (EC), cortex and hippocampus and were selected manually by a trained expert following the Paxinos atlas. The volume measurements of structural MRI images were obtained using the ITK-SNAP software.^19^

### Blood analysis in patients

The results from full blood counts with leucocyte differential at Day 7 post-TBI, measured by a standard clinical automated haematology analyser (Department of Pathology, Cambridge University Hospital, UK) as part of routine clinical care, were acquired from patients’ medical records. Matching data were also gathered from patients admitted to the intensive care unit (ICU) with viral pneumonitis as a non-TBI ICU control group. Population-based reference ranges were available for each parameter. To assess the effect of age on leucocyte responses, two age-defined subgroups from the TBI patients were examined to mirror the mouse experiments: ‘Adult’ patients (18–45 years old), and ‘Aged’ patients >70 years old. Demographics of the patient cohorts are reported in Table 1.

**Table 1:** Demographics of the patient cohorts

	Whole TBI cohort	ICU	TBI	TBI	*P*-value
		Control	Adult	Aged	
Cohort size	68	20	21	23	
Sex (male)	45	10	17	15	0.24
(%)	(66%)	(50%)	(81%)	(65%)	
Age (years) Median (IQR)	59 (38–72)	66 (56–71)	28 (24–36)	76 (72–83)	<0.0001
GCS on admission Median (IQR)	7 (4–9)	Not applicable	6 (4–7)	9 (5–10)	0.03

### Flow cytometry

Mice were deeply anaesthetized; the spleen was removed and immediately frozen in ice-cold RPMI medium. Single-cell spleen suspensions were subjected to red blood cell lysis buffer, washed twice with PBS containing 2% foetal bovine serum (FBS) and resuspended in the same solution before the split in 2 aliquots and blocking with 5 μg/ml rat anti-mouse CD16/32 (BD Biosciences) and 0.5 mg/ml normal rat serum for 10 min on ice to reduce non-specific antibody binding. Viability staining was performed with Zombie UV Fixable Viability Kit (BioLegend) for 20 min at room temperature (RT). The cell suspensions were then washed and stained following cell surface anti-mouse antibody (1/200) combinations for 30 min at 4°C. Immune cell antibody combination: CD45 APC (30-F11, Biolegend), CD8 FITC (53-6.7, Biolegend), CD4 PE/Dazzle (RM4-5, Biolegend), CD11b Brilliant Violet 605 (M1/70, Biolengend), CD11c Alexa fluor 700 (N418, Biolegend), NK1.1 PE (PK136, Biolegend), Ly6C PerCP (HK1.4, Biolegend), Ly6G Alexa fluor 780 (1A8, Biolegend) and MHCII e450 (AF6-120.1, Invitrogen). B-cell antibody combination: CD45 APC (30-F11, Biolegend), B220 PerCP (RA3-6B2, Invitrogen), CD19 eFluor 450 (1D3, Invitrogemn), IgD PE/Dazzle (11-26c.2a, Biolegend), CD3 Brilliant Violet 605 (17A2, Biolegend), CD5 Brilliant Violet 510 (53-7.3, Biolengend) and CD11b Brilliant Violet 650 (M1/70, Biolengend). Flow cytometry data collection was performed on an LSR Fortessa flow cytometer (BD Biosciences), and data were analysed using the FlowJo software (Treestar, v10.6). The gating strategy is shown in Supplementary Fig. 1. Monocytes were identified as CD45^+^/CD11b^+^/Ly6G^low^/Ly6C^int^, T cells as CD45^+^/Cd11b^−^/CD3^+^, B cells as CD45^+^/CD11b^−^/B220^+^, Eosinophils as CD45^+^/CD11b^+^/Ly6G^low^/Ly6C^Hi^, Neutrophils as CD45^+^/CD11b^+^/Ly6G^Hi^/Ly6C^int^ and Dendritic cells as CD45^+^/CD11b^+^/CD11c^+^.

### RNAseq analysis

Mice were transcardially perfused with 20 ml ice-cold PBS. Skull caps, with intact dura mater, were dissected and placed in an RNA-later buffer for RNAseq analysis. The dura mater from each animal was dissected out in ice-cold PBS, RNA extraction was performed using RNeasy Plus Micro Kit (Qiagen Cat No. 74034) and the RNA library using TruSeq stranded total RNA sample preparation (Illumina Cat No. 20020596). Libraries were then sequenced on a Hiseq 4000 sequencer (Ilumina) by Genewiz. Further details are given in the GEO series record. Following sequencing, the data were demultiplexed to give individual fastq files using the Casava (Illumina). The Fastq files were assessed for quality control purposes using FASTQC. The Fastq files were aligned to the murine genome (mm10) using Hisat2. All further analysis was carried out using the R statistical environment. A table of gene counts was produced using the featureCounts function within Rsubread and normalization and differential gene expression analysis was carried out using DESeq2. For GSEA, genes were ranked by the inverse of the *P*-value with the sign of the log fold change and then ran against the hallmarks database within MSigDB using the GSEA program from the broad with the pre-ranked option. ssGSEA was carried out using normalized counts from DESeq2. All batch information, Fastq files, count tables and differential expression results are included in the GEO/EGA series record.

### Histology

Mice were deeply anaesthetized with ketamine (20 mg, intraperitoneally, IP) and medetomidine (0.2 mg, IP) then transcardially perfused with 30 ml of PBS 0.1 mol/l (pH 7.4), followed by 60 ml of chilled paraformaldehyde (PFA 4%) in PBS. The brains were carefully removed from the skull, post-fixed in 4% PFA in PBS overnight (o/n) at 4°C, dehydrated with 30% sucrose in 0.1 mol/l PBS for 24 h at 4°C, frozen in *n*-pentane for 3 min at −45°C and stored at −80°C. Serial coronal brain sections (20 μm) were cut on a cryostat (+1 to −4 mm from bregma) at 200 μm intervals for histological analysis.

Mice dedicated to meningeal histology were perfused with 30 ml of PBS. Skull caps, with intact dura mater, were dissected and maintained overnight in PAF 2%. The fixed tissue was washed with PBS 3 times for 5 min per wash. Under a dissecting microscope, the dura mater was dissected out carefully with forceps from the skull cap, leaving the meninges intact, in a Petri dish containing PBS. Meninges were then stored at 4°C in PBS in a 24-well plate until they were used for immunostaining.

Neuronal cell loss and white matter damage were examined on Nissl^12,20^ and luxol fast blue (LFB)^13^ stained sections, respectively. Two coronal sections per mouse, −0.4 and −1.6 mm from bregma, were acquired at 20× magnification with an Olympus BX-61-VS microscope, interfaced with VS-ASW-FL software (Olympus, Germany). The acquisition was done over 10 μm thick stacks, with 2 μm step size. The different focal planes were merged into a single stack by the mean intensity projection to ensure consistent focus throughout the sample.

For neuronal counts, the ipsilateral (il) cortex was analysed over an area 0.6 mm deep from the edge of the contusion. A corresponding area of the contralateral (cl) hemisphere and sham animals was analysed. The degree of neuronal loss was calculated as the percentage of neurons in the ipsilateral over the contralateral hemisphere, normalized over the acquisition area. Images were analysed with the Fiji software and segmentation was used to distinguish neurons from glia on the basis of cell size.^13^

For myelin density calculated in il-EC, the mean optical density (OD) of LFB-stained sections was measured with Fiji over an area 0.4 mm deep from the edge of the contusion. To account for variable lighting and staining intensity, normalized myelin density was calculated as (OD_white matter_ – OD_background_)/(OD_cortex_ – OD_background_).^21^

#### Immunohistochemistry of mouse meningeal whole mounts

The meningeal tissue was immersed in a blocking and permeabilization solution containing 2% normal rat serum, 2% normal donkey serum, 1% bovine serum albumin (BSA), 0.1% Triton X-100 and 0.05% Tween in PBS for 1 h RT. Then the meninges were stained with cell surface anti-mouse antibodies in 1% BSA, 0.5% Triton X-100 in PBS at 4°C overnight. A combination of the following primary antibodies was used for immunohistochemistry (IHC): CD45 Brilliant violet 650 (30-F11, 1/100, Biolegend), B220 Alexa fluor 647 (RA3-6B2, 1/25, BD Pharmigen), CD3ε Alexa fluor 488 (145-2C11, 1/25, Biolegend), CD11b PE (M1/70, 1/50, Invitrogen), Collagen IV (Rb pAb to Coll IV, 1/100, Abcam). Following the primary staining, meninges were washed three times in PBS for 5 min per wash, RT. For the collagen IV non-conjugated antibody, anti-rabbit IgG (Dnk pAb to RB IgG, 1/250, Abcam) was applied in PBS for 2 h at RT secondary staining, then meninges were washed 3 times in PBS for 5 min per wash at RT. Using a paintbrush, the dura mater was flattened on a glass slide and mounted with Fluoromount-G, with Dapi (Invitrogen). Slides were kept in the dark at 4°C before imaging.

Tile scan imaging of whole-mount meninges or brain slices was carried out on either a Leica SP8 confocal microscope equipped with five detectors, nine laser lines (405, 442, 458, 488, 496, 514, 561 and 633 nm) and four objectives (10×, 20×, 40× and 63×).

#### Brain IHC

Heat-induced antigen retrieval was done by incubating sections in sodium citrate buffer pH 6.0 for 20 min at 80°C. After cooling to RT, slices were washed with PBS and endogenous peroxidases were inactivated with 1% hydrogen peroxide for 10 min at RT.

**GFAP:** blocking was achieved by applying 10% foetal calf serum, 0.5% Triton in PBS for 1 h, followed by overnight incubation 4°C with the primary antibody mouse anti-GFAP (1:2000; Chemicon). After washing with PBS, sections were incubated with secondary antibody solution for 1 h at RT, followed by incubation with the Vectastain Elite ABC reagent (Vector Laboratories, Burlingame, CA, USA).

**IBA-1:** staining was done using an EXPOSE Rabbit-specific HRP/DAB detection IHC kit (AbCam). Slices were incubated in the blocking solution for 10 min, followed by incubation with primary antibody rabbit anti-mouse IBA-1 (1:200; Wako, Osaka, Japan) in a solution containing 0.2% Triton, 2% normal horse serum, in PBS for 2 h at RT. After washing with PBS, sections were incubated with horseradish peroxidase-conjugate secondary antibody solution for 15 min at RT.

Immunoreactivity was tested using DAB as a chromogen (Vector Laboratories, Burlingame, CA, USA). For each staining, negative controls were run in parallel and in each case, no staining was observed.

#### Brain immunofluorescence

After antigen retrieval slices were blocked in 0.5% Triton-X100, 10% FBS in PBS. Primary antibodies for mouse anti-GFAP (1:2000), rabbit anti-aquaporin 4 (AQP 4, 1:1000; Millipore, clone AB3594), rabbit anti-IBA1 (1:200), rat anti-myelin binding protein (MBP, 1:200; Chemicon) or rabbit anti-non-phosphorylated neurofilaments (SMI-32; 1:1000; Abcam) were incubated overnight at 4°C. Fluorescent secondary antibodies (1:500, Alexa 488 conjugate of goat anti-mouse and Alexa 594 conjugate of goat anti-rabbit) were used. Negative controls were run without the primary antibodies.

#### Acquisition and quantification of IHC and immunofluorescence

Three brain coronal sections including the core lesion (0.4, −1.6, −2.8 mm from bregma)^12^ per marker for each mouse were acquired at 20× with an Olympus BX-61 Virtual Stage microscope (for IHC) or Nikon A1 confocal scan unit managed by the NIS elements software [for immunofluorescence (IF)] with 10% overlap for stitching, *z*-axis 10 μm (step size 2 μm). For each coronal section, ROIs for the contused cortex, pericontusional cortex and EC (Supplementary Fig. 2) were selected by a trained operator in accordance with atlas references.^22^ Contused cortex ROIs were drawn at 0.2 mm depth from the contusion edge over three coronal sections. Pericontusional cortex ROIs were designed of 0.2 mm depth from the contused cortex over three coronal sections. The spared il-EC and the whole cl-EC were drawn over two coronal sections (−1.6, −2.8 mm from bregma).

Images were analysed using the Fiji software. For IHC markers, the immunostained area was expressed as the percentage stained area. For IF markers, the mean grey value was quantified as a measure of fluorescence intensity. The degree of white matter damage was calculated as the ratio of SMI-32 to MBP fluorescence intensity.^23^

### Gene expression analysis

Ipsilateral cortical areas were dissected out 3 or 7 days after surgery, immediately frozen on dry ice and stored at −80°C until analysis. Total RNA was extracted with a PureLink RNA Mini Kit (Thermofisher). Samples were treated with DNase (Life Technologies) and reverse-transcribed with random hexamer primers using Multi-Scribe Reverse Transcriptase (Life Technologies). Real-time reverse transcription PCR was done with *RPL27* as the housekeeping gene. Relative gene expression was determined by ΔΔ*C_t_* method. Data are expressed as the log2-fold difference over the sham adult group. Genes and primer sequences are listed in Supplementary Table 1.

### Statistical analysis

All graphs show the mean ± standard error of the mean (SEM). GraphPad Prism 8 was used for statistical analyses. Data were analysed by two-way ANOVA for repeated measures in case of longitudinal assessments (behavioural tests) or by two-way ANOVA (MRI, brain histological analyses and log2 values of gene expression data) followed by Tukey’s or Sidak *post hoc* tests. Spleen flow cytometry data and dura mater immune cells counting were analysed by an unpaired *t*-test. *P*-values of <0.05 were considered statistically significant. Assumptions of normality were checked using the D’Agostino–Pearson omnibus test. Outliers were identified using the ROUT method and excluded from the analysis. Comparison of leucocyte populations between human TBI patients and population-based reference ranges was performed using one-sample *t*-tests. Differences between human TBI and viral pneumonitis patients and between adult and aged TBI patients were analysed using Mann–Whitney U-tests.

### Data availability

The data that support the findings of this study are available from the corresponding author upon reasonable request.

## Results

### Aged TBI mice show greater sensorimotor deficits than adult mice across injury severities

Simple neuroassessment of asymmetric impairment (SNAP) scores detected an injury-severity effect 1, 3 and 6 weeks post-TBI, with sTBI inducing greater sensorimotor deficits than mTBI in adult (*P* < 0.01) and aged (*P* < 0.001) mice (Fig. 1B). An age effect was found at each timepoint, with aged mice showing greater sensorimotor deficits across TBI severities (mTBI: *P* < 0.001; sTBI: *P* < 0.001). In addition, mTBI in the aged mice caused a similar degree of functional impairment compared with sTBI in adult mice.

Sensorimotor deficits were additionally tested using Neuroscore. At all time points, aged sham mice performed worse than adult (*P* < 0.05), indicating age-related impairment in sensorimotor performance. All TBI mouse groups performed significantly worse than their age-matched sham controls (*P* < 0.05). No differences were found between adult and aged TBI mice in the first week after trauma (Fig. 1C). At 3 and 6 weeks, aged showed greater sensorimotor deficits than adult mice following mTBI (*P* < 0.001), but not sTBI, possibly because of a floor effect in the latter.

### Aged TBI mice show decreased recovery potential compared with adult mice despite lower contusion volume

Contusion volume, recorded by T2w-MRI, was greater in sTBI than mTBI (*P* < 0.001), with aged mice having lower contusion volumes than adult mice (Fig. 1D). The spared tissue at the contusion edge, with Nissl staining, showed similar neuronal death across ages and severities (Fig. 1E).

When the SNAP score was correlated with the contusion volume, a direct correlation was observed for adult (Pearson correlation *r* = 0.859, *P* < 0.001) and aged TBI mice (Pearson correlation *r* = 0.659, *P* < 0.01) at 1 week (Fig. 1F), but at 6 weeks, only aged mice showed a significant correlation (Pearson *r* = 0.823, *P <* 0.001). These data indicate time-dependent recovery of function exclusively in adults, suggesting reduced plasticity in aged mice.

### Aged sTBI mice have more ipsilateral white and grey matter abnormalities than adult mice

MRI undertaken at 5 weeks post-TBI showed an injury-dependent reduction in FA in il-EC (*P* < 0.001, Fig. 1H). TBI increased AD and RD only in aged mice (Fig. 1I and J). *Post hoc* analysis indicated both AD (*P* < 0.001) and RD (*P* < 0.01) were significantly higher in aged than adult sTBI mice. DTI analysis in the cl-EC showed no differences between adult and aged mice with the same injury severity (Supplementary Fig. 3B–D).

MD in ipsilateral grey matter areas (Fig. 1K and L) showed a severity-dependent increase only in the aged mice, where significant increases were only seen in the hippocampus (Fig. 1L). No differences were found between adult and aged mice across severities for MD in contralateral grey matter brain areas (Supplementary Fig. 3E and F).

No age-related differences were observed after mTBI.

### TBI induces an acute age-dependent systemic immune activation in humans and mice

**Figure 2:**
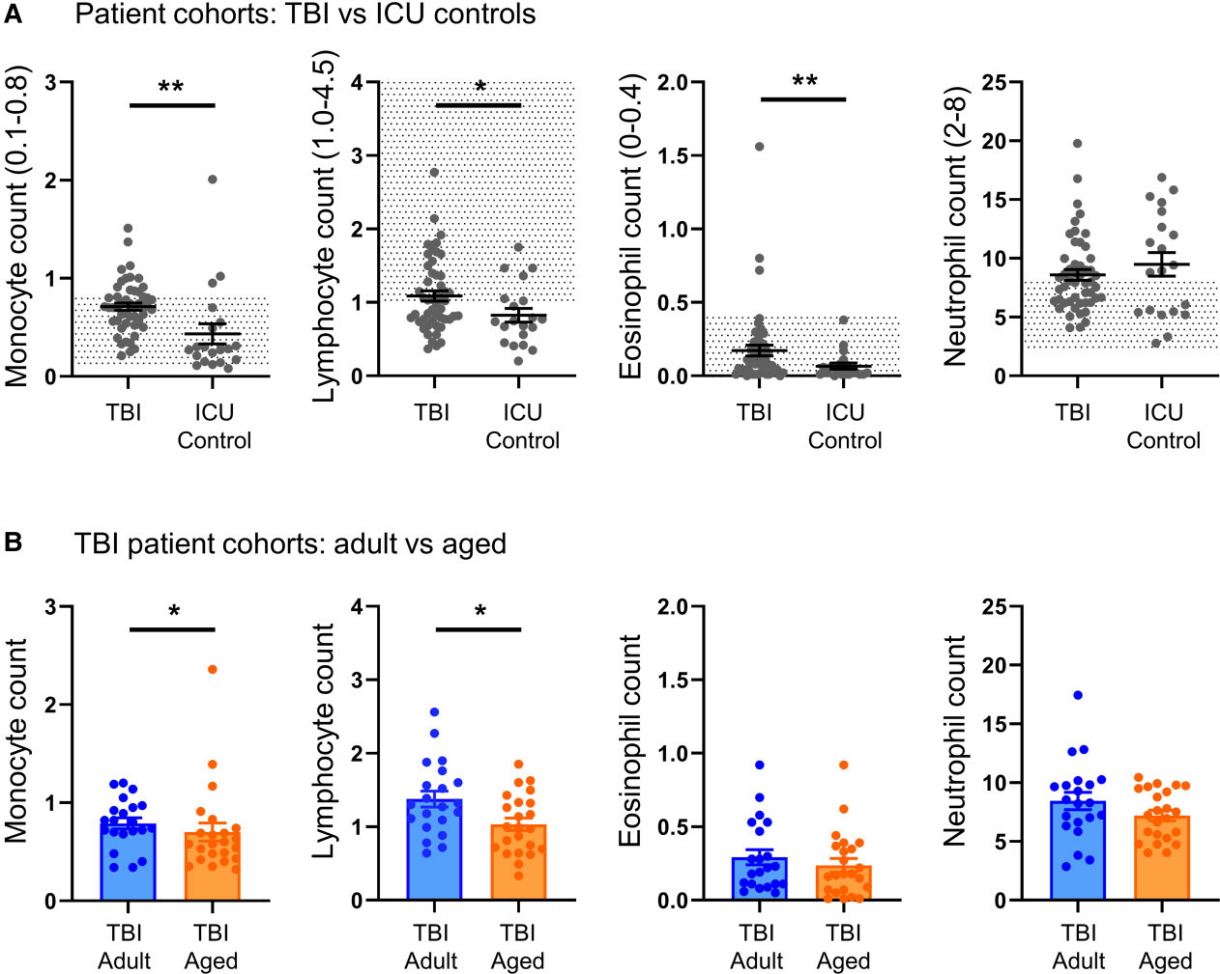
**Human blood immune response.** (**A**) Blood levels of immune cells in ICU controls and TBI patients 7 days after admission. Population-based reference ranges are shown as dotted areas. (**B**) Comparison between leucocyte populations in adult (<45 years) and aged (>70 years) TBI patients at 7 days post-TBI. Data are mean ± SEM. Each data point refers to a single patient. Mann–Whitney U-test, * *P* < 0.05, ** *P* < 0.01.

Compared with population-based reference ranges, 68 patients with mTBI to sTBI (admission GCS <13, Table 1) exhibited a significant increase in neutrophils (*P* < 0.0001), monocytes (*P* < 0.0001) and eosinophils (*P* < 0.01) and a significant reduction in lymphocytes (*P* < 0.0001) at Day 7 post-TBI (Fig. 2A), with no sex-related differences (Supplementary Fig. 4). The increase in monocytes and eosinophils was significantly greater in the TBI group than in the 20 non-TBI ICU control patients (*P* < 0.0001 for both comparisons), whereas the reduction in lymphocyte count was less marked (*P* < 0.001 compared with ICU controls) (Fig. 2A). Within the TBI cohort, an age effect on immune response was present, with significantly lower monocyte and lymphocyte counts in patients older than 70 compared with those younger than 45 years at 7 days post-TBI (monocytes *P* < 0.05 and lymphocytes *P* < 0.05, Fig. 2B), and the suggestion of a similar (but non-significant) trend in neutrophil and eosinophil counts.

**Figure 3:**
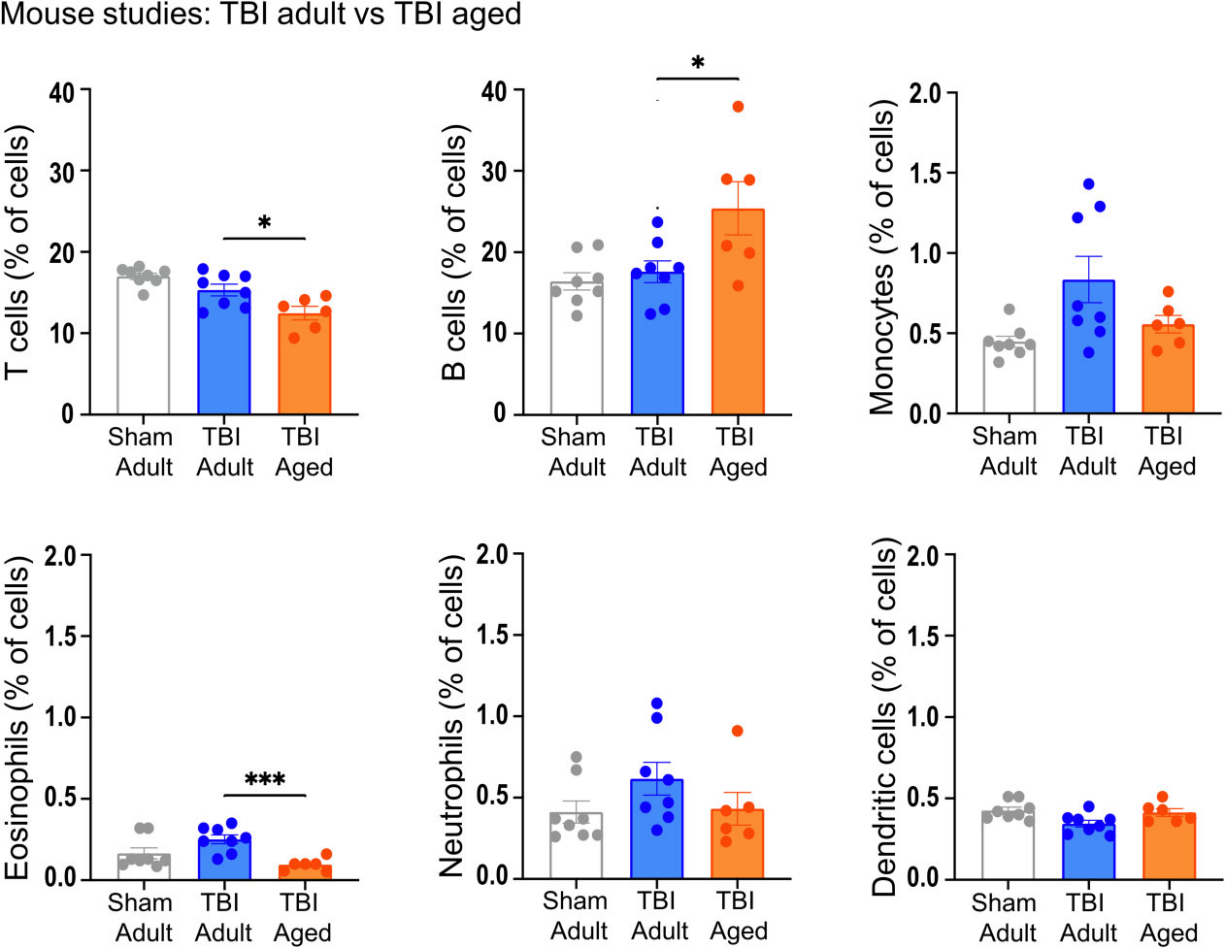
**Mouse splenic immune response.** Flow cytometry analyses of immune cells present in the spleen, 3 days after injury. Comparison between adult versus aged severe TBI mice. Sham adult mice are shown as a reference. Data are mean ± SEM. Each data point refers to a single animal. Mann–Whitney U-test, *P < 0.05, ***P < 0.001.

After experimental TBI, aged sTBI mice showed a tendency towards reduction of monocytes, an increase in B cells (*P* < 0.05) and a reduction of T cells and eosinophil count in the spleen (*P* < 0.05) compared with adult, with no major effects of age in the other immune cell populations (Fig. 3).

### TBI associated with peripheral monocytosis induces pathway-specific meningeal immune response with increased macrophage infiltration in aged sTBI

**Figure 4:**
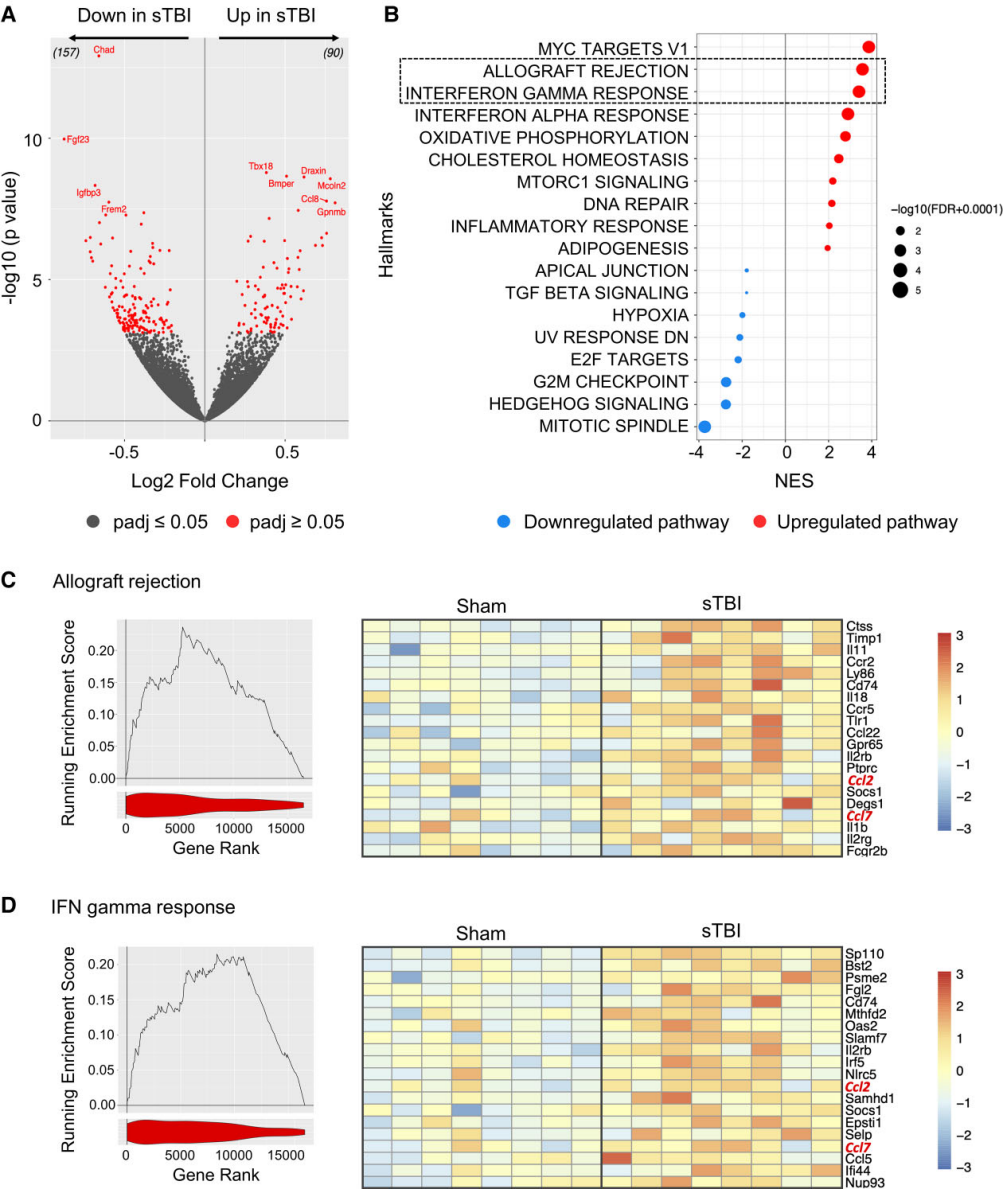
**Analysis of gene expression in the meninges after sham and sTBI.** (**A**) Volcano plot indicates changes in gene expression between meninges from mice with sTBI compared with sham controls, 6 weeks post-injury. Red dots indicate significantly differentially expressed genes (adjusted *P*-value <0.05) and the top 10 genes ranked by significance have been labelled. The numbers into brackets correspond to genes up or down represented. (**B**) GSEA against the hallmarks set of pathways for the analysis presented in **A**. Only significantly enriched pathways have been shown. The size of the point inversely correlates with the level of significance of the enrichment, the colour, the direction and the position correspond to the normalized enrichment score (NES). (**C and D**) Left panels. Individual enrichment plots showing the enrichment for allograft rejection (**C**) or IFN-γ (**D**) pathways. The line indicates the running enrichment score. The violin plot under the graph indicates the position of the gene set within the ranked list of genes from the differential expression in **B**. Right panels. Heatmap showing the top 20 genes from the leading edge of the pathways in the corresponding enrichment plot.

There is an increasing appreciation that the meninges house a variety of immune cells that have the potential to respond to local and systemic perturbations. To interrogate meningeal immunity in TBI, we performed bulk RNA sequencing of the dura mater in sham and TBI mice. More than 200 genes were differentially expressed in TBI dura, including 90 upregulated and 157 downregulated genes found in TBI mice compared with sham 6 weeks post-injury (Fig. 4A). Notably, *Ccl8*, a leucocyte-recruiting chemokine, was among the most upregulated genes in TBI dura (Fig. 4A). Gene-set enrichment analysis (GSEA) demonstrated significant upregulation of immune-related pathways, including the ‘allograft rejection’ and ‘interferon gamma response’ gene-sets that included *Il1b* and monocyte-recruiting chemokines *Ccl2* and *Ccl7* (Fig. 4B–D).

**Figure 5:**
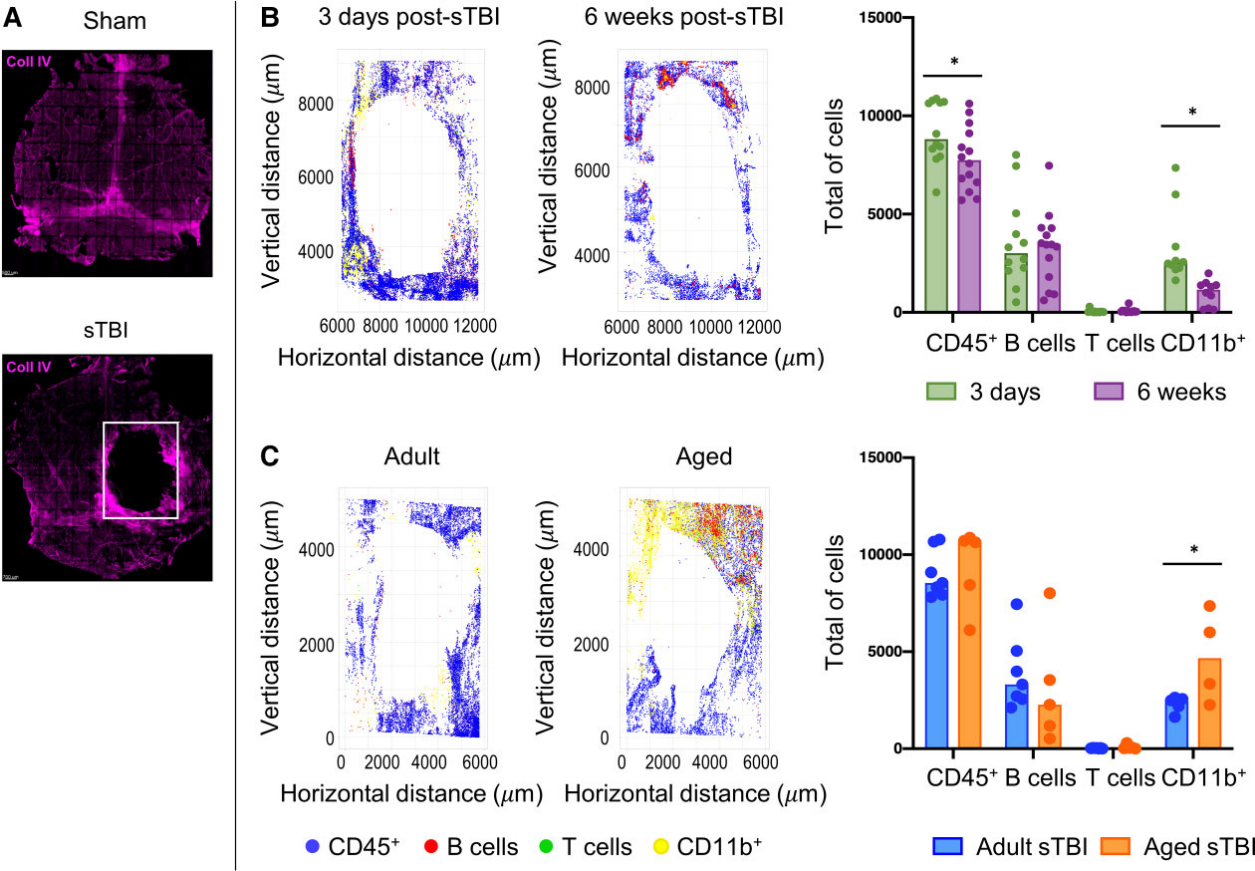
**Immunostaining analysis in the meninges after sham and sTBI.** (**A**) Representative IHC images of meninges from sham and sTBI mice stained with collagen IV. (**B**) IHC and immune cells counting by confocal microscopy around the injury site after sTBI. Comparison of the immune cell numbers between 3 days and 6 weeks post-sTBI. (**C**) IHC and immune cells counting by confocal microscopy around the injury site after sTBI. Comparison of the immune cell numbers 3 days post-sTBI between adult and aged mice. Data are mean ± SEM. Each data point refers to a single animal. Two-way ANOVA followed by Tukey *post hoc*, *P < 0.05.

Confocal imaging of dural whole mounts showed a marked accumulation of CD45^+^ immune cells around the injury site, with CD11b^+^ myeloid cells prominent at 3 days post-TBI (Fig. 5B). When comparing adult with aged mice 3 days post-TBI, there was a significant increase in CD11b^+^ myeloid cell recruitment to the border of the dural injury in aged mice (Fig. 5C).

### Ageing increases TBI-induced reactive astrogliosis and microglial activation in mice

**Figure 6:**
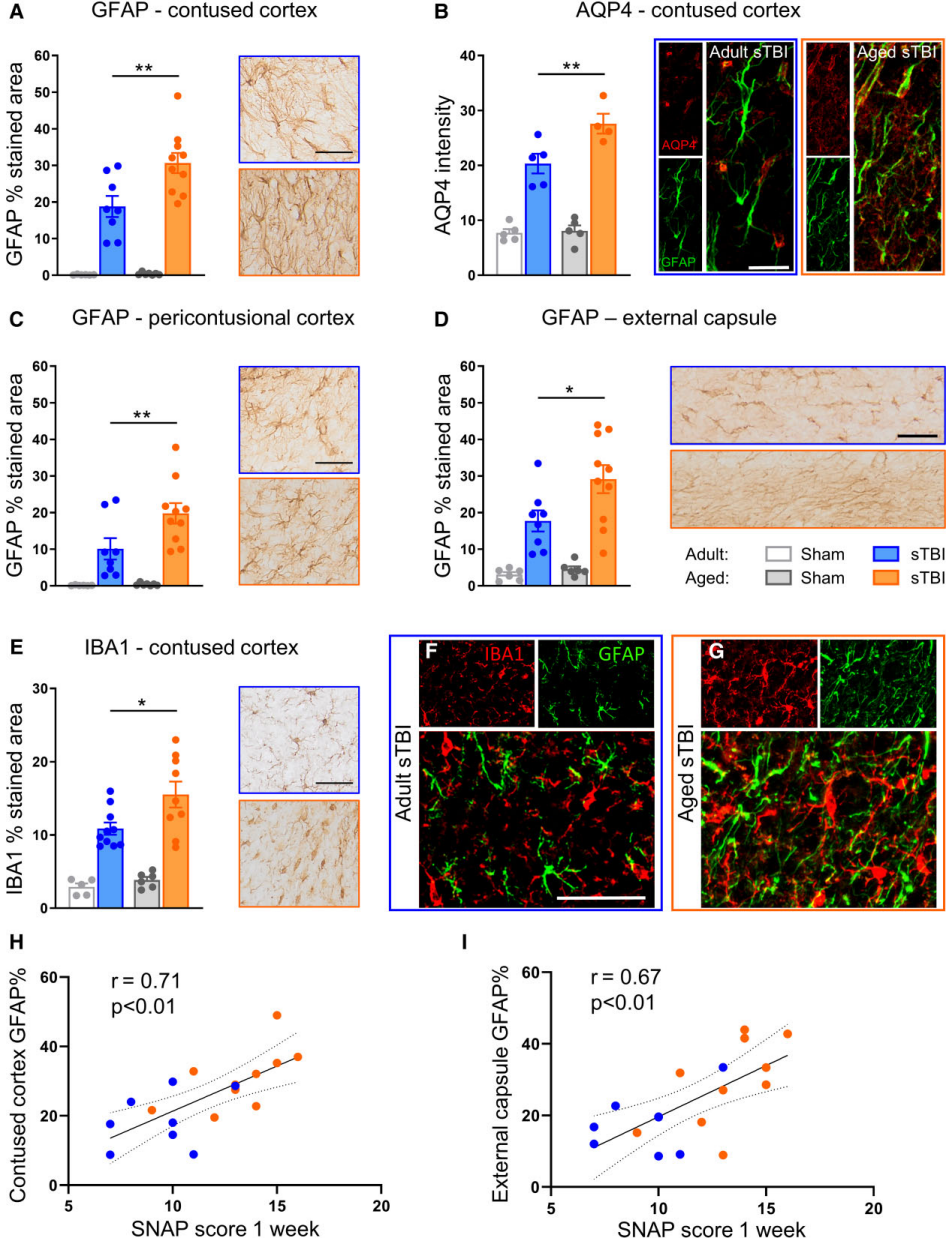
**Glial activation in ipsilateral areas 6 weeks post-TBI.** Representative IHC images of GFAP-stained area and quantification in the contused (**A**) and pericontusional (**C**) cortex and EC (**D**). (**B**) Representative IF images of AQP4 (red) and GFAP (green) and AQP4 quantification in the contused cortex. (**E**) Representative IHC images of the IBA1-stained area and quantification in the contused cortex. Representative IF images of IBA1 (red) and GFAP (green) in the contused cortex of adult (**F**) and aged (**G**) sTBI mice. Bar 50 μm (**A**, **C**, **D–F**) or 25 μm (**B**). Correlation between SNAP test at 1 week and GFAP-stained area at 6 week post-injury in the contused cortex (**H**) and EC (**I**). (**A**–**E**) Data are mean ± SEM. Each data point refers to a single animal. Two-way ANOVA followed by Sidak multiple comparison test, *P < 0.05, **P < 0.01. (**H** and **I**) Pearson correlation.

Reactive astrogliosis was present at the contusion edge (Fig. 6A) after sTBI, with aged mice showing greater GFAP labelling than adults (*P* < 0.01). In the same ROI, there was more labelling and loss of perivascular polarization of AQP4 in aged than adult mice (*P* < 0.05, Fig. 6B). Widespread and more pronounced reactive astrogliosis in aged compared with adult sTBI mice was also observed in the pericontusional cortex (Fig. 6C) and il-EC (Fig. 6D). In the contralateral hemisphere, GFAP staining showed no age-related differences in the cortex (Supplementary Fig. 5A) or cl-EC (Supplementary Fig. 5B) after sTBI. Reactive astrogliosis was less widespread and pronounced after mTBI, with no age effects in any of the area analysed (Supplementary Fig. 5C–E).

Microglial/macrophage (M/m) activation, identified using IBA1 staining, was significantly greater at the contusion edge in aged sTBI mice (*P* < 0.05, Fig. 6E) while there were no differences between adult and aged sTBI mice in the pericontusional cortex (Supplementary Fig. 6A), il-EC (Supplementary Fig. 6B) or contralateral areas (Supplementary Fig. 6E and F). il-EC damage was also analysed by quantifying myelin density by LFB (Supplementary Fig. 7A–C) and myelin integrity by SMI-32/MBP ratio (Supplementary Fig. 7D–G). Both measurements showed greater damage after sTBI, with no age-related differences.

Notably, in sTBI mice, the functional outcome at 1 week (evaluated by SNAP test) was strongly correlated with the percentage of GFAP-stained area in both the contused cortex (Pearson *r* = 0.71) and EC (Pearson *r* = 0.67) at 6 weeks (Fig. 6H and I). The correlation was less marked with IBA1 staining in the contused cortex (Pearson *r* = 0.52, Supplementary Fig. 6C) and absent in the EC (Pearson *r* = 0.24, Supplementary Fig. 6D).

### sTBI induces a more prominent acute reactive astrogliosis in aged mice

**Figure 7:**
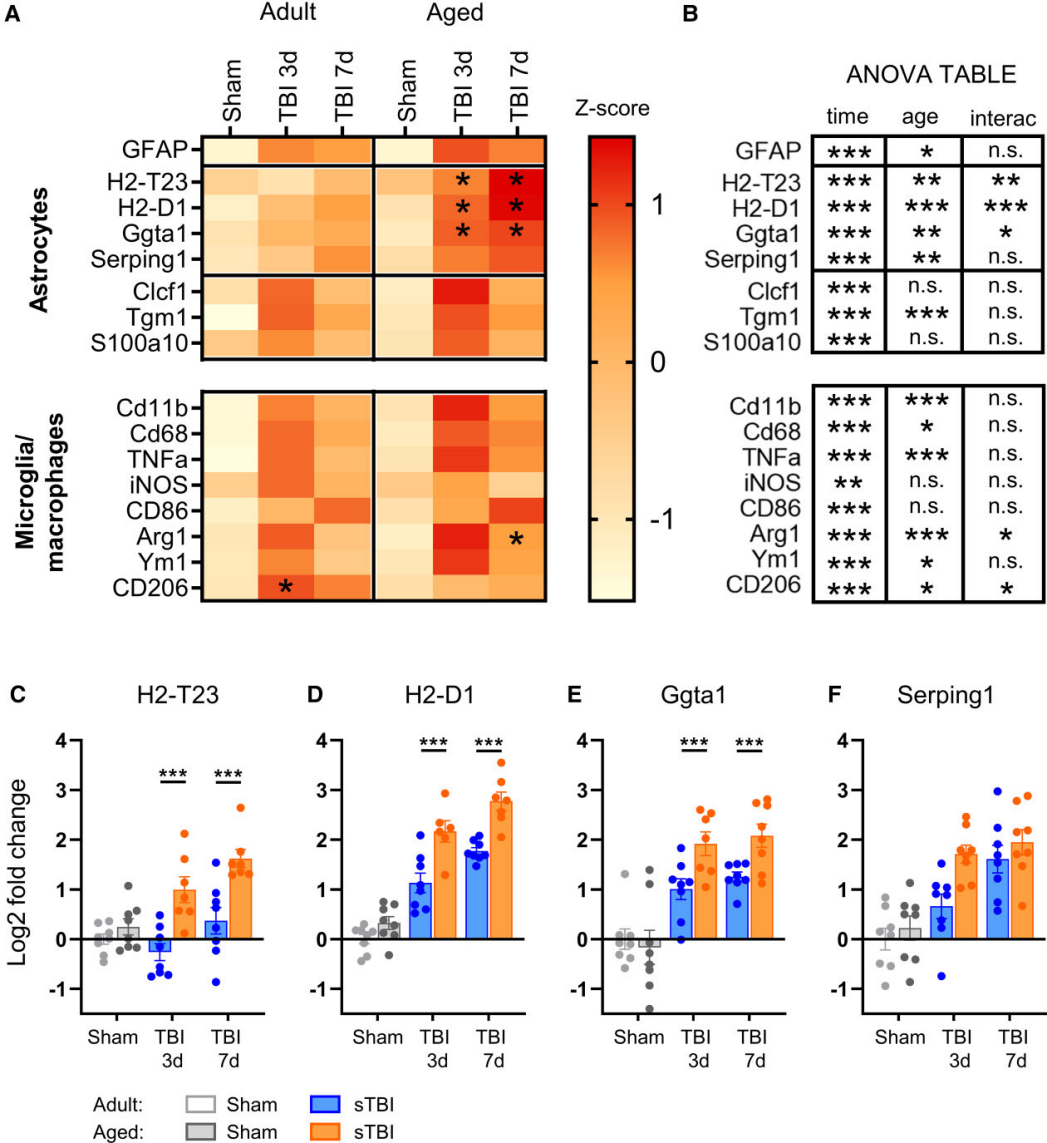
**Age-related changes in acute gene expression of glial markers.** (**A**) Gene expression of adult and aged animals subjected to sham or severe TBI and sacrificed at 3 or 7 days. Each rectangle shows the mean *Z*-score of log2 gene expression of each group. (**B**) Summary statistic of log2 values. (**C–F**) Graphs representing dot plots of LPS-induced related genes. All data are log2-fold change relative to adult sham values. Data are mean ± SEM. Each data point refers to a single animal. Log2 values are analysed by two-way ANOVA followed by Sidak’s multiple comparison test. *P < 0.05, **P < 0.01, ***P < 0.001.

To further investigate glial activation, we examined gene expression in the il-cortex 3 and 7 days after sTBI. Ageing (*P* < 0.05) and sTBI (*P* < 0.001) both increased the expression of astrocytes (*GFAP*) and M/m (*CD11b* and *CD68*, *P* < 0.05) pan markers (Fig. 7A and B and Supplementary Fig. 8A, E and F).

We then evaluated the expression of astrocytic-related genes previously found altered during neuroinflammation and previously classified as A1 (LPS-induced) or A2 (stroke-induced).^24,25^ Compared with adult, aged sTBI mice showed elevated expression of the LPS-induced genes (*H2-T23*, *H2-D1*, *Ggta1*; *P* < 0.05, Fig. 7C–F) up to 7 days. In contrast, stroke-induced genes (*Clcf1*, *Tgm1*, *S100a10*, Fig. 5A and B and Supplementary Fig. 8B–D) showed a transient increase at 3 days that was reduced by 7 days.

The evaluation of M/m-related genes showed acute activation of *TNFα*, *iNOS*, *CD86*, *Arg1*, *Ym1* and *CD206* markers after sTBI mice (Fig. 7A). Of note, *Arg1* dropped at 7 days in adult sTBI mice compared with the aged group (*P* < 0.01), whereas *CD206* expression was lower in this latter at all the time points considered (Fig. 7A and B and Supplementary Fig. 8J–L).

## Discussion

Our data show expected behavioural deficits, imaging abnormalities and systemic, meningeal and cerebral immune responses following experimental TBI, which broadly scaled with injury severity. However, we also show that aged mice showed clear differences from adult mice in each of these metrics. These differences were underpinned by greater DTI abnormalities in aged compared with the young adult mice, and associated with an altered systemic, meningeal and brain tissue immune responses.

The worse functional outcome in aged mice could not be explained by a greater contusion volume or greater neuronal cell loss in the spared cortex. Aged mice had smaller contusion volumes than adult mice, but greater deficits, such that SNAP scores, in aged mTBI mice were similar to those seen in adult sTBI mice. The more severe initial behavioural deficits in aged mice were also more persistent. While there was a positive correlation between the contusion volume and functional deficits at acute stages (1 week post-TBI) in both adult and aged mice, at later times (6 weeks post-TBI), the correlation persisted only in aged mice, indicating time-dependent recovery of function in adult that appeared lacking in aged mice with a similar injury.

To clarify the microstructural white matter changes associated with an age effect, we undertook DTI at 5 weeks post-TBI, which showed increased abnormalities in the aged compared with adult TBI mice. In agreement with previous reports in both human^26^ and experimental^13^ studies, we found a TBI-dependent decrease in FA, but found no interaction between age and trauma. In contrast, AD and RD values in aged sTBI mice were significantly greater than adults. Clinical findings in TBI show increases of both AD^27,28^ and RD^28–30^ in several white matter tracts at chronic stages. Disentangling the precise biological processes contributing to these DTI results is complicated, since they may be driven by greater demyelination or greater glial activation, both of which could explain the increase in the mean diffusivity and FA reduction in white matter.^31,32^ Our data support a role of reactive astrogliosis in this context, since white matter regions, where DTI was abnormal in sTBI mice but not in mTBI, showed greater astrogliosis only in sTBI aged animals, while there was no significant age effect of TBI on myelin as assessed by LFB or SMI32/MBP staining.

In grey matter, we found similar numbers of neurons in the pericontusional cortex across ages and TBI severities. This finding, together with an increase in microgliosis in the contused cortex, and more widespread reactive astrogliosis supports the notion that the outcome differences between adult and aged mice are not due to greater tissue loss at the site of injury, but to differences in focal and systemic neuro-inflammatory response.

The acute systemic immune response is a key determinant of the secondary brain injury after TBI.^33^ In TBI patients, we found a pattern of increased monocyte, neutrophil and eosinophil counts, as well as reduced lymphocyte counts compared with population-derived reference values. When compared with non-TBI ICU controls, the monocyte, lymphocyte and eosinophil count was significantly higher following TBI. Interestingly, within TBI patients, a significantly lower monocyte and lymphocyte counts in patients older than 70 compared with those younger than 45 years was detected. The heterogeneity of clinical TBI and the impact of comorbidities in older patients make it hard to clarify the precise pathological mechanisms involved in these changes. Preclinical models enable to explore the consequences of TBI in a controlled setting in which age is the only variable, with no confounding factors. In line with patient data from our group, Ritzel *et al*.^7^ found acute accumulation of myeloid cells in the spleen 3 days post-TBI in young but not aged mice with no age-related differences in sham. We limited our analysis to TBI cohorts and confirmed the tendency towards an increase in myeloid cell populations (including eosinophils, monocytes and neutrophils) in the spleen of young TBI compared with aged mice. We also extended previous findings by exploring T- and B-cell lymphocytes subsets. Aged TBI mice had lower T cells compared with adult TBI mice. Peripheral immune cells have access to the meninges and parenchyma with a role in the modulation of brain damage and functional recovery.^34,35^ Brain-penetrating T cells may limit neuronal damage after mechanical injury^36^ while regulatory T-cell depletion may induce reactive astrogliosis and worsen outcomes after TBI.^37^ On the contrary, we observed a higher accumulation of B cells in aged compared with adult sTBI mice. Since a reduction of B cells in the periphery (including the spleen) has been shown in naïve mice during ageing, the increase we observed is likely specific to TBI.^38^ One possible interpretation is that TBI exacerbates age-associated B cells accumulation leading to an increased tendency for inflammatory responses, and an increased production of autoantibodies.^39^

By profiling meningeal immunity, we found a clear induction of ‘allograft rejection’ and ‘interferon gamma’ immune-related pathways. This included *Il1b* and monocyte-recruiting chemokines *Ccl2* and *Ccl7* upregulation which have a role in recruitment and activation of tissue macrophage/microglial and T cells and expand what previously shown acutely after meningeal compression in a model of mild TBI.^40^ Our data show that infiltration/proliferation of CD11b cells in the meninges around the injury site was more robust in aged compared with adult sTBI mice. It has been demonstrated that after mild TBI, different subsets of myeloid cells are involved in meningeal repair, participating either in dead-cell removal or supporting angiogenesis.^40^ Nevertheless, an elevation of CD11b^+^ cells has been reported in pathological conditions.^40,41^ Intriguingly, we have recently shown that B cells have immunoregulatory effects on meningeal myeloid cells that include also the regulation of IFN-γ signalling in the meninges^41^ thus suggesting that the elevation of CD11b in the meninges of aged TBI could also be mediated by splenic B cells and INF-γ-mediated signalling.

Recent data point to a crucial role of the meningeal lymphatic system in modulating inflammation in the CNS^42^ and that meningeal lymphatic drainage function could be targeted to mitigate neuroinflammation in aged mice subjected to closed head TBI.^10^ Our data indicate that sTBI with a dural breach is associated with a marked local inflammatory response, with the production of monocyte- and leucocyte-recruiting chemokines driving the accumulation of myeloid cells, and that this was more prominent in aged mice. We should acknowledge that we limited our analysis on the effect of ageing on the infiltration/proliferation of immune cells in the meninges around the contusion border. Thus, no conclusion can be drawn on the isolated contribution of ageing to the observed changes.

We also found a marked increase in inflammatory genes in aged mice at acute times, pointing to a role for systemic lymphocytes in exacerbating reactive astrogliosis in the elderly. An effect of age on glial activation up to 2 weeks post-TBI has already been shown for microglia^10,43,44^ and astrocytes.^10,45^ Our results extend these findings by documenting increased and widespread reactive astrogliosis up to 1 month post-injury with a strong correlation with functional outcomes. Astrocytes contribute to interstitial homeostasis by controlling water and ion gradients via AQP4, a water channel localized predominantly in astrocytic end-feet surrounding the microcirculation.^46^ After TBI, there is an increase in AQP4, which loses its perivascular topology and accumulates in the astrocyte soma and in non-perivascular regions.^47,48^ This altered distribution prevents glymphatic clearance of interstitial solutes (such as mis-aggregated proteins) post-injury.^49^ We found higher levels of AQP4 in aged compared with adult mice, with a clear loss in perivascular polarization, supporting the hypothesis of impaired glymphatic clearance in the elderly, possibly contributing to chronic neurodegenerative events.

Acute gene expression analysis in the ipsilateral cortex confirmed histological data with greater *GFAP* expression in aged than adult TBI mice, confirming previous findings.^45^ Interestingly, ageing was associated with an early and persistent increased expression of *H2-T23*, *H2-D1* and *Ggta1* genes, which have been previously found elevated after LPS-induced neuroinflammation^24,25,50^ or human neurodegenerative disease.^24^ In agreement with previous data,^43,51^ M/m pan markers *CD11b* and *CD68* were increased by both sTBI and ageing. Nevertheless, the age-related M/m response to trauma showed mixed results with higher *Arg1* and lower *CD206* expression in aged compared with adult sTBI. A potential limitation of the present analysis is that gene expression was performed in bulk tissue. However, these data are in line with histological evidence of increased reactive astrogliosis in aged TBI mice and point to a major role of reactive astrocytes,^52^ in the age-related vulnerability to TBI. Due to astrocytic involvement in the regulation of blood brain barrier (BBB) permeability^53^ and leucocytes recruitment to the CNS,^54^ we speculate that the exacerbation of reactive astrogliosis in aged sTBI mice may influence meningeal and systemic immune reactivity that, in turn further aggravates neuroinflammation and neurological outcome.

## Conclusions

We found age-related differences in behavioural recovery, imaging findings and pathological processes after TBI that have clear clinical relevance. Increasing life expectancy and active lifestyles raise the risk of accidental falls in the elderly population,^55^ who are more vulnerable to brain damage, and have worse outcomes and greater mortality than younger individuals.^6^ We found a slower and less complete recovery from functional deficits after TBI in the elderly. Aged mice that sustained a mTBI showed similar impairment to adult mice with a sTBI, suggesting a synergistic role of age and injury severity on TBI outcome. Behavioural differences are underpinned, in aged mice, by DTI-MRI abnormalities in white matter structures, and accompanied by a dysregulated systemic, meningeal and brain tissue immune response, as well as widespread reactive astrogliosis with indications for a proinflammatory signature. Further studies are needed to investigate the functional correlate of reactive astrocytes identified in TBI aged mice and their impact on disease progression in terms of BBB damage, overactivation of systemic/meningeal immunity and worsening of neurological outcome.

## Supplementary Material

fcac036_Supplementary_Data

## Abbreviations

AD =: axial diffusivity
AQP4 =: aquaporin 4
BBB =: blood brain barrier
BSA =: bovine serum albumin
cl =: contralateral
DTI =: diffusion tensor imaging
EC =: external capsule
EPI =: echo planar imaging
FA =: fractional anisotropy
FBS =: foetal bovine serum
GFAP =: glial fibrillary acidic protein
GSEA =: gene-set enrichment analysis
IBA1 =: ionized calcium binding adaptor molecule 1
ICU =: intensive care unit
IF =: immunofluorescence
IHC =: immunohistochemistry
il =: ipsilateral
IP =: intraperitoneally
IQR =: interquartile range
LFB =: luxol fast blue
MBP =: myelin binding protein
MD =: mean diffusivity
mTBI =: moderate TBI
n.s. =: not significant
NES =: normalized enrichment score
OD =: optical density
PFA =: paraformaldehyde
RD =: radial diffusivity
RF =: radio frequency
ROIs =: regions of interest
RT =: room temperature
SEM =: standard error of the mean
SMI-32 =: non-phosphorylated neurofilaments
SNAP =: simple neuroassessment of asymmetric impairment
sTBI =: severe TBI
T2w =: T_2_ - weighted
TBI =: traumatic brain injury
TE =: echo time
TR =: repetition time
