# Supplementary material for: Ageing is associated with maladaptive immune response and worse outcome after traumatic brain injury
Source: Brain Commun. 2022 Feb 16;4(2):fcac036. doi: 10.1093/braincomms/fcac036 (PMC8947244; doi:10.1093/braincomms/fcac036)
Supplement: fcac036_Supplementary_Data [file fcac036_supplementary_data.pdf]

Aging is associated to maladaptive immune response and worse outcome after traumatic brain injury

Supplementary materials

Supplementary table 1

**Gene**	**NCBI**	**Forward primer**	**Reverse primer**
RPL27	NM_011289	TCATGAAACCCGGGAAAGT	GAGGTGCCATCGTCAATGT
GFAP	NM_001131020.1	GAAAACCGCATCACCATTCC	TCGGATCTGGAGGTTGGAGA
H2-T23	NM_010398	GGACCG CGAATGACATAG C	GCACCTCAGGGTGACTTC AT
H2-D1	NM_010380	TCCGAGATT GTAAAGCGTGAAGA	ACAGGGCAGTGCAGGGAT AG
Ggta1	NM_001308300	GTTTTGTTGCCTCTGGGTGT	GTGAACAGCATGAGGGGTTT
Serping1	NM_009776	ACAGCC CCCTCTGAA TTC TT	GGATGCTCTCCAAGTTGCTC
Clcf1	NM_001310038	CTTCAATCCTCCTCGACTGG	TACGTCGGAGTTCAGCTGTG
Tgm1	NM_001161715	CTGTTGGTCCCGTCCCAAA	GGACCTTCCATTGTGCCT GG
S100a10	NM_009112	CCTCTGGCTGTGGACAAAAT	CTGCTCACAAGAAGCAGTGG
CD11b	NM_010562.2	GAGCAGCACTGAGATCCTGTTTAA	ATACGACTCCTGCCCTGGAA
CD68	AK002264	GGATTGGATTGAGGAAGGAACTG	GCCGCATGGCAGAGATG
TNFα	NM_013693.2	AGACCCTCACACTCAGATCATCTTC	TTGCTACGACGTGGGCTACA
iNOS	NM_001313922.1	CAAGCACCTTGGAAGAGGAG	AAGGCCAAACACAGCATACC
CD86	NM_019388.3	GTTACTGTGGCCCTCCTCCTT	CTGATTCGGCTTCTTGTGACATA
Arginase-1	NM_007482.3	CATGGGCAACCTGTGTCCTT	TCCTGGTACATCTGGGAACTTTC
Ym1	NM_009892.2	TCTGGTGAAGGAAATGCGTAAA	GCAGCCTTGGAATGTCTTTCTC
CD206	NM_008625.2	CCCAAGGGCTCTTCTAAAGCA	CGCCGGCACCTATCACA

Supplementary table 1. *Primers used for real-time reverse transcription polymerase chain reaction*. National Center for Biotechnology Information (NCBI).

Supplementary Figure 1

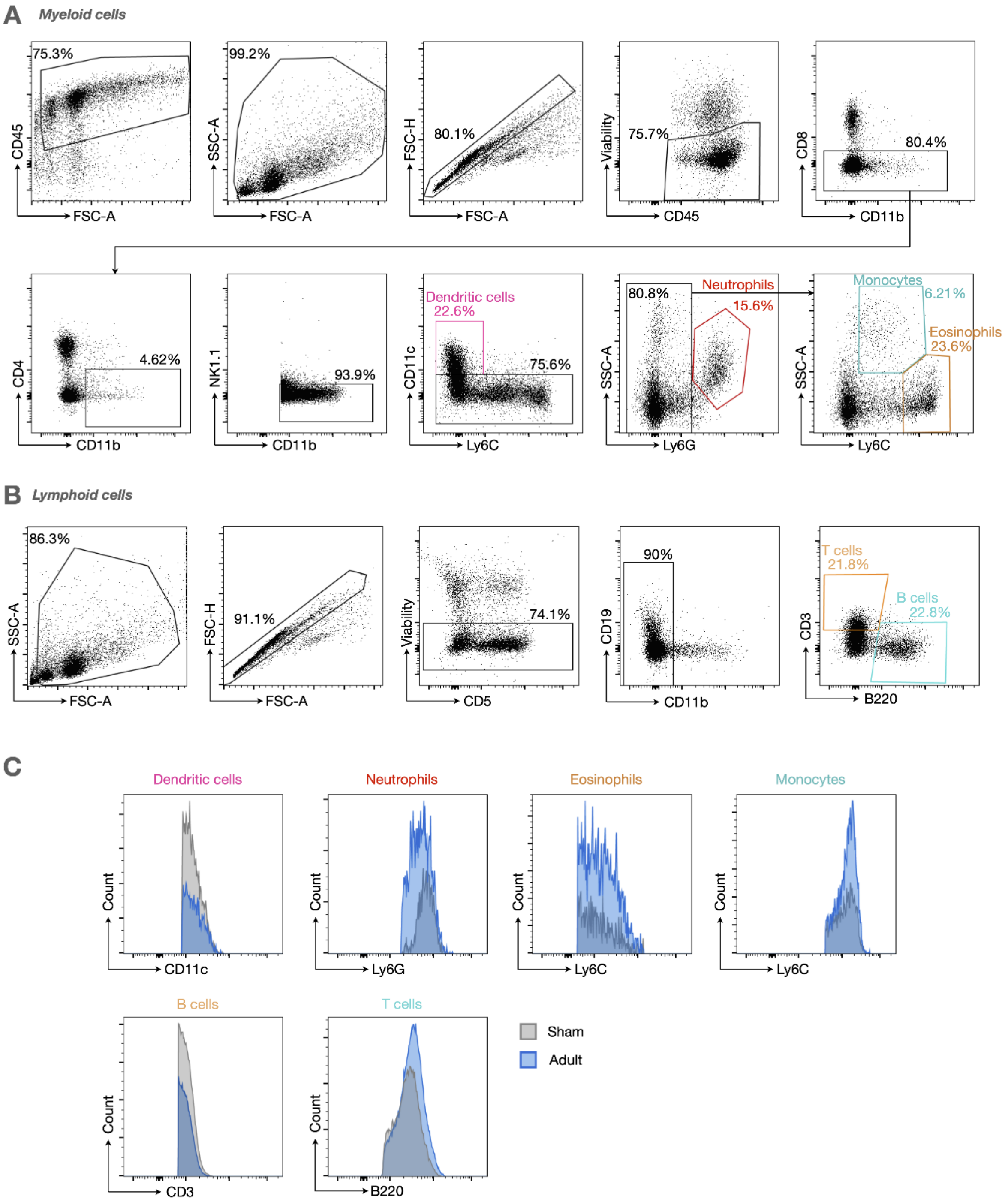

Supplementary Figure 1. Gating strategy for the different immune populations in the spleen. (A) Gating strategy for myeloid cells: Dendritic cells (pink gate), Neutrophil cells (red gate), Eosinophil cells (orange gate) and Monocyte cells (blu gate). (B) Gating strategy for lymphoid cells: T cells (orange gate) and B cells (blue gate). (C) Mean Fluorescence Intensity (MFI) profiles for each type of highlighted immune cells. Comparison between sham (grey profile) and TBI adult (blue profile) samples.

Supplementary Figure 2

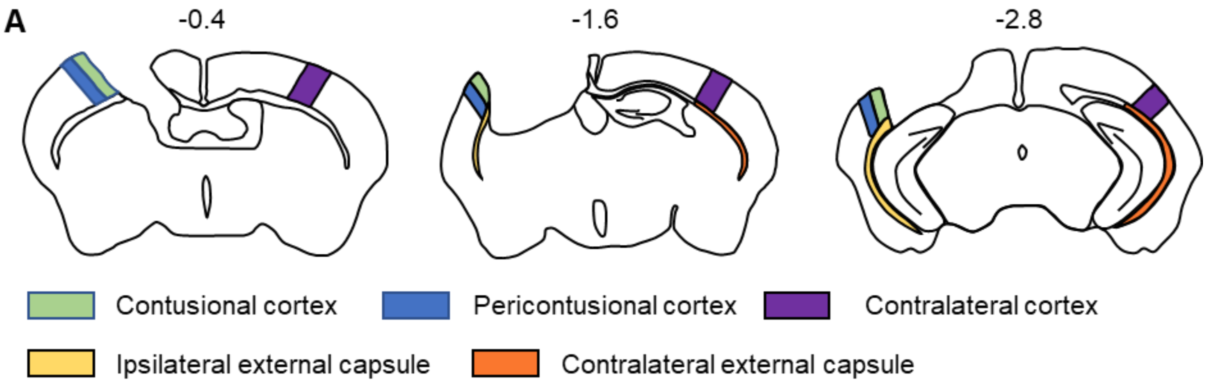

Supplementary Figure 2. Schematic drawings representing ROIs for IHC analysis.

Supplementary Figure 3

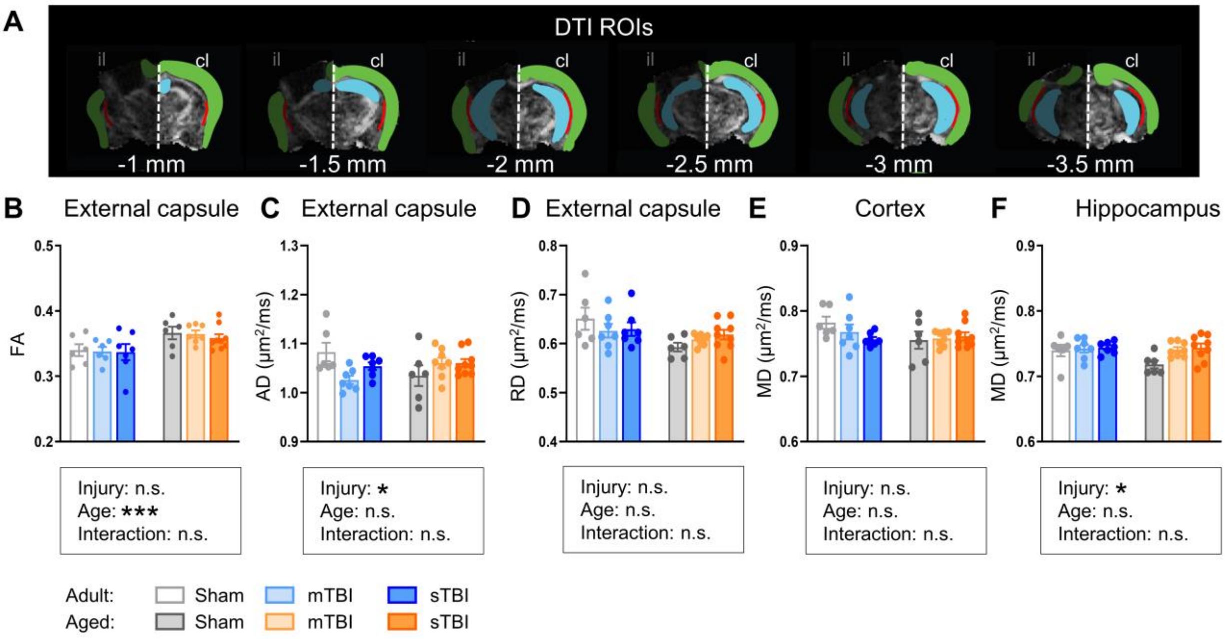

Supplementary Figure 3. *Quantitative analysis of diffusion tensor imaging (DTI) parameters in the contralateral white and gray matter.* Drawings represent the rostrocaudal ROI selection for external capsule (red), cortex (green) and hippocampus (blue) structures (A). Quantification of fractional anisotropy (B), axial diffusivity (C) and radial diffusivity (D) in the external capsule or mean diffusivity in the cortex (E) or hippocampus (F). Data are mean ± SEM. Each data point refers to a single animal. Two way ANOVA with Tukey's test. Significant group effects of age, injury and interaction are shown in each box. *p<0.05, **p<0.01, ***p<0.001.

Supplementary Figure 4

A

Characteristic	Male *median (IQR)*	Female *median (IQR)*	P-Value
Age	55 (36-73)	64 (52-73)	0.30
GCS on admission	6 (4-9)	7 (4-10)	0.80
Lymphocyte count	1.14 (0.81-1.54)	1.18 (0.98-1.46)	0.66
Eosinophil count	0.22 (0.11-0.35)	0.22 (0.11-0.38)	0.88
Monocyte count	0.72 (0.54-0.91)	0.68 (0.52-0.8)	0.49
Neutrophil count	7.23 (5.59-9.66)	7.50 (5.83-9.85)	0.62

B

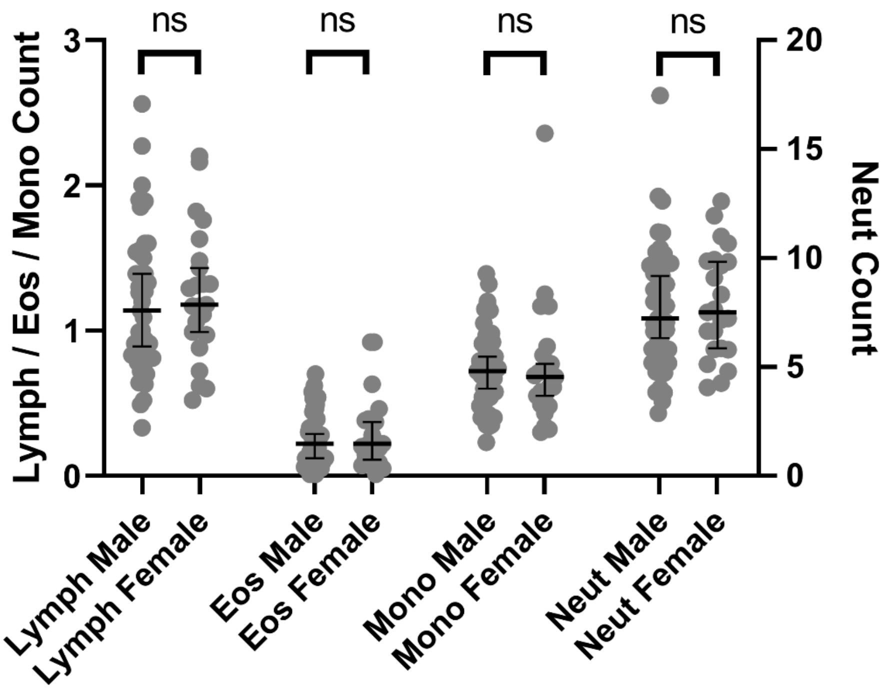

Supplementary Figure 4. *Sex related differences of human blood immune response.* **(A)** Table reporting demographics of the TBI patient cohort divided per sex. **(B)** Comparison of blood levels of leucocyte populations between male and female TBI patients at 7 days after admission.

Supplementary Figure 5

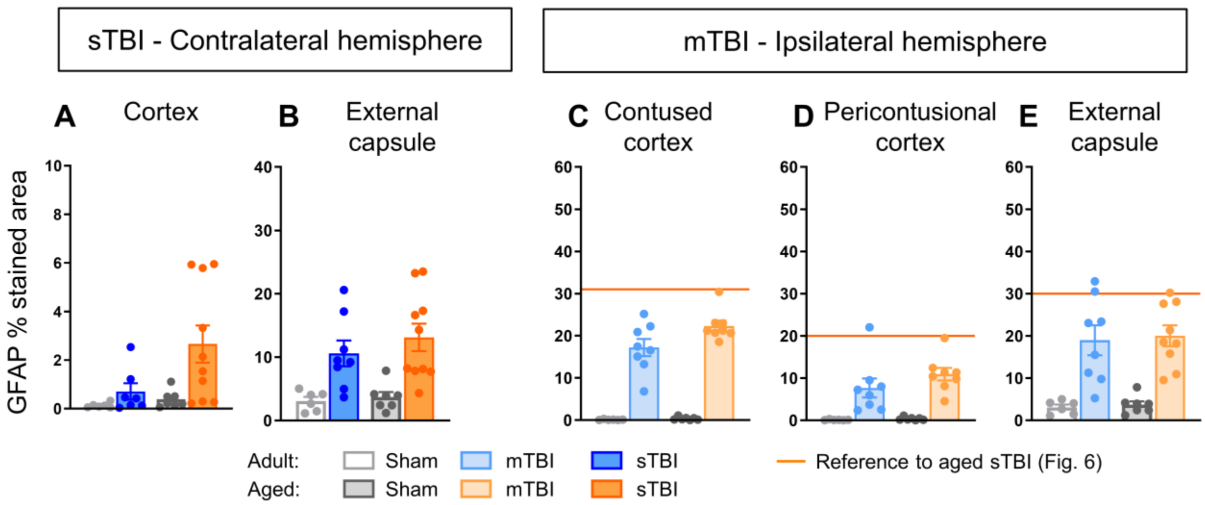

Supplementary Figure 5. *GFAP histological analysis 6 weeks post injury.* Quantification of GFAP staining in sTBI mice in the contralateral cortex (A) and external capsule (B) and in mTBI mice in the ipsilateral contused cortex (C), pericontusional cortex (D) and external capsule (E). Data are mean ± SEM. Each data point refers to a single animal. Two way ANOVA.

Supplementary Figure 6

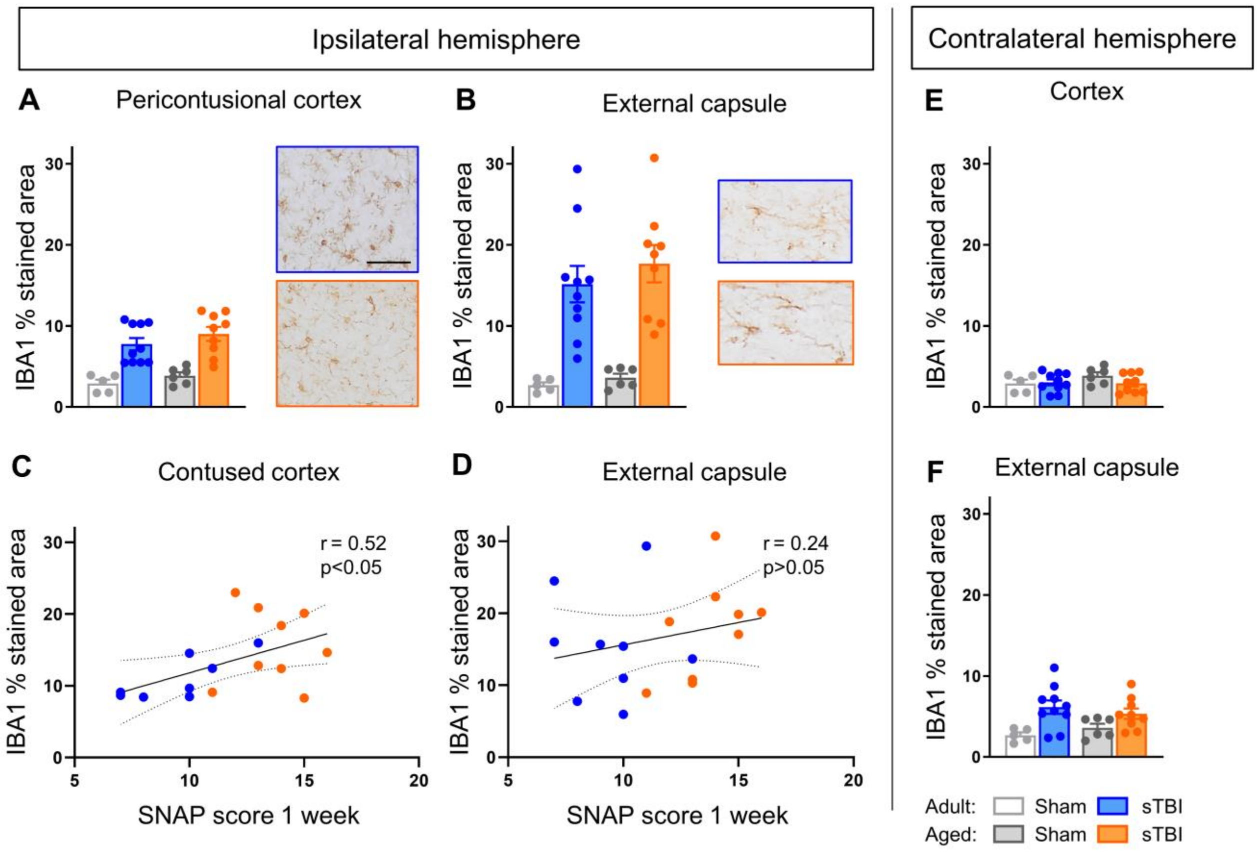

Supplementary Figure 6. *IBA1 histological analysis 6 weeks post injury.* Quantification of IBA1 staining in sTBI mice in the ipsilateral pericontusional cortex (A) and external capsule (B). Correlation between SNAP test at 1 week post-injury and IBA1 stained area in the contused cortex (C) and external capsule (D).

Quantification of IBA1 staining in sTBI mice in the contralateral cortex (E) and external capsule (F). Data are mean ± SEM. Each data point refers to a single animal. Two way ANOVA.

Supplementary Figure 7

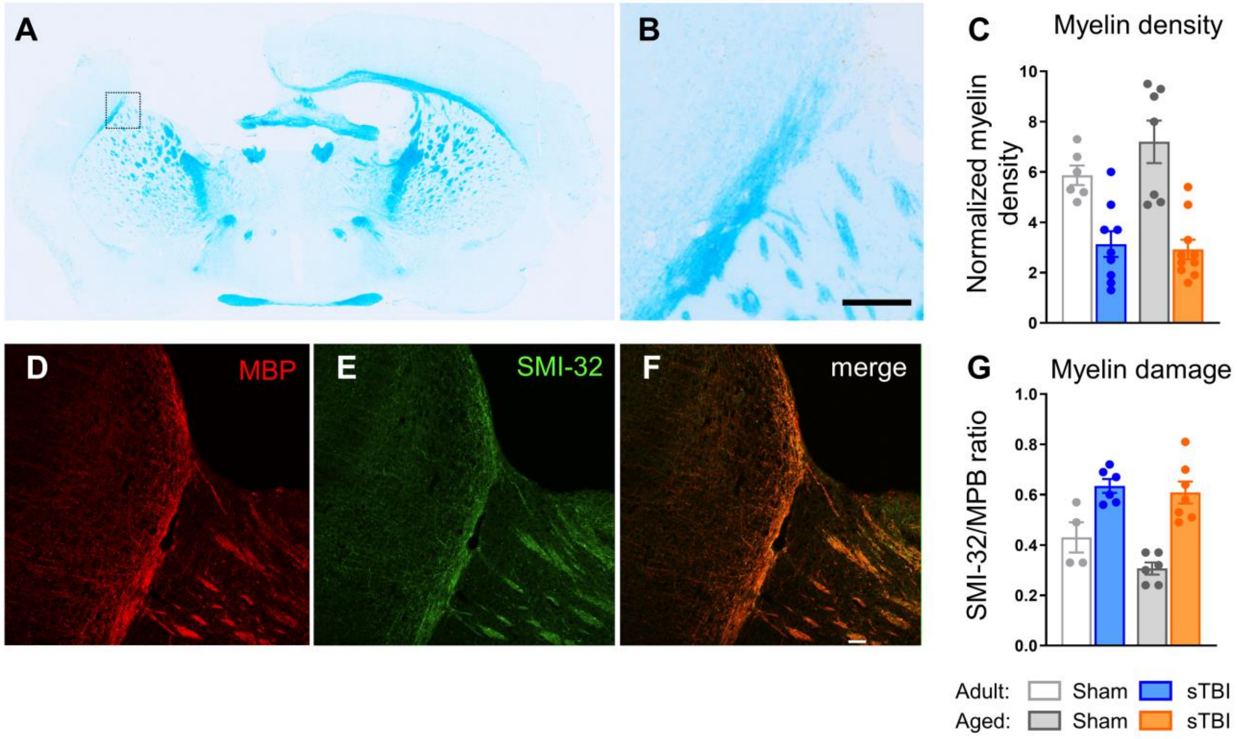

Supplementary Figure 7. *White matter histological analysis 6 weeks post injury.* Quantification of luxol fast blue intensity (A-C) and SMI32/MPB ratio (D-G) in the ipsilateral external capsule. Data are mean ± SEM. Each data point refers to a single animal. Two way ANOVA.

Supplementary Figure 8

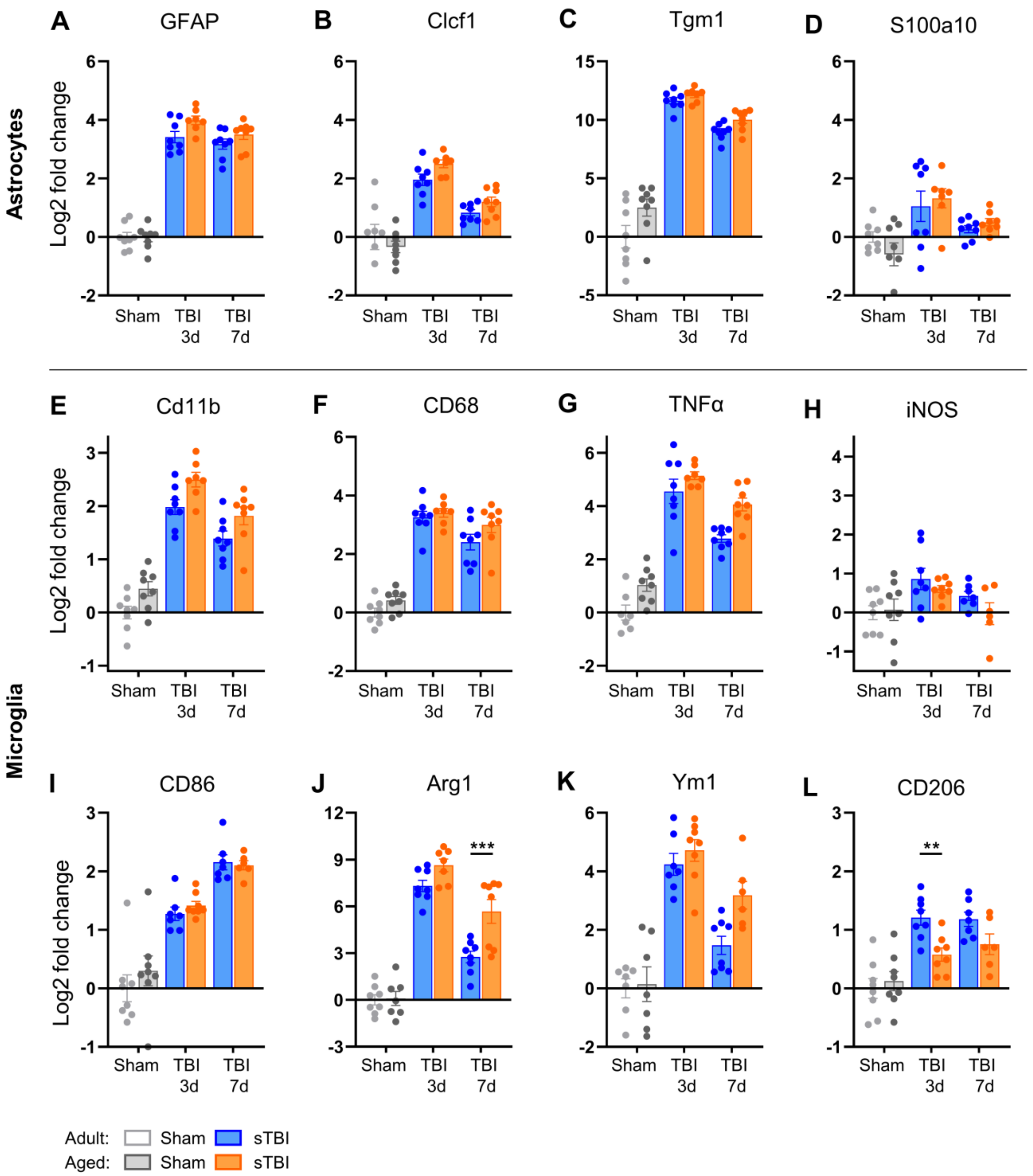

Supplementary Figure 8: *Age-related changes in acute gene expression of glial markers.* Gene expression of adult and aged animals subjected to sham or severe TBI and euthanized at 3 and 7 days. All data are the log2-fold difference form adult sham values. Data are mean ± SEM. Each data point refers to a single animal. Log2 values were analysed by two-way ANOVA followed by Sidak multiple comparison test. **p<0.01, ***p<0.001.
